# Super-High-Frequency Bulk Acoustic Resonators Based on Aluminum Scandium Nitride for Wideband Applications

**DOI:** 10.3390/nano13202737

**Published:** 2023-10-10

**Authors:** Wentong Dou, Congquan Zhou, Ruidong Qin, Yumeng Yang, Huihui Guo, Zhiqiang Mu, Wenjie Yu

**Affiliations:** 1 School of Information Engineering, Southwest University of Science and Technology, Mianyang 621010, China; douwentong@mails.swust.edu.cn; 2 State Key Laboratory of Functional Materials for Informatics, Shanghai Institute of Microsystem and Information Technology, Chinese Academy of Sciences, Shanghai 200050, China; zhoucq@mail.sim.ac.cn (C.Z.); radonqin@mail.sim.ac.cn (R.Q.); casan@mail.sim.ac.cn (W.Y.); 3 Center of Materials Science and Optoelectronics Engineering, University of Chinese Academy of Sciences, Beijing 100049, China; 4 Shanghai Engineering Research Center of Energy Efficient and Custom AI IC, School of Information Science and Technology, ShanghaiTech University, Shanghai 201210, China; yangym1@shanghaitech.edu.cn

**Keywords:** bulk acoustic wave, film bulk acoustic resonator, aluminum scandium nitride, effective electromechanical coupling coefficient, spurious modes

## Abstract

Despite the dominance of bulk acoustic wave (BAW) filters in the high-frequency market due to their superior performance and compatible integration process, the advent of the 5G era brings up new challenges to meet the ever-growing demands on high-frequency and large bandwidth. Al_1-x_Sc_x_N piezoelectric films with high Sc concentration are particularly desirable to achieve an increased electromechanical coupling (*K_t_*^2^) for BAW resonators and also a larger bandwidth for filters. In this paper, we designed and fabricated the Al_1-x_Sc_x_N-based BAW resonators with Sc concentrations as high as 30%. The symmetry of the resonance region, border frame structure and thickness ratio of the piezoelectric stack are thoroughly examined for lateral modes suppression and resonant performance optimization. Benefiting from the 30% Sc doping, the fabricated BAW resonators demonstrate a large effective electromechanical coupling (*K_eff_*^2^) of 17.8% at 4.75 GHz parallel resonant frequency. Moreover, the temperature coefficient of frequency (TCF) of the device is obtained as −22.9 ppm/°C, indicating reasonable temperature stability. Our results show that BAW resonators based on highly doped Al_1-x_Sc_x_N piezoelectric film have great potential for high-frequency and large bandwidth applications.

## 1. Introduction

With the rapid development of 5G and WiFi technology, the evolution of wireless communication systems towards high speed and large capability requires radio frequency (RF) filters to have high frequency, wide bandwidth and compact size [1]. Emerging 5G and WiFi 6E/7 spectrums adopt frequencies in super-high-frequency (SHF, 3–30 GHz) band and bandwidth above 10%, such as n79 (4.4–5.0 GHz) and UNII1-3 (5.15–5.85 GHz). Acoustic filters, including surface acoustic wave (SAW) and bulk acoustic wave (BAW) filters, show promising potential in these new bands for mobile applications. Limited by the relatively low surface acoustic velocity and electro-migration damage of sub-micrometer inter-digital transducers (IDTs), it is very challenging for SAW filters to operate above 3 GHz [2]. As a result, BAW filters based on AlN piezoelectric film currently dominate the applications above 2.5 GHz due to its high acoustic velocity of thickness-extensional waves, high thermal conductivity and compatibility with mainstream complementary metal-oxide-semiconductor (CMOS) process. Moreover, the working frequency of BAW filters is proportional to the thickness of AlN film, which is favored by device miniaturization at higher frequencies.

On the other hand, the relatively low intrinsic longitudinal piezoelectric response and electromechanical coupling coefficient (*K_t_*^2^) of AlN piezoelectric film limit the fractional bandwidth of as-fabricated BAW filters to less than 3%. Rare earth element doping is regarded as the most effective method to improve the piezoelectric response of AlN film [3]. Among them, scandium (Sc) doping has been attracting enormous attention since Akiyama M fabricated an Al_1-x_Sc_x_N piezoelectric film with a piezoelectric coefficient up to four times larger than that of pure AlN in 2009 [4]. Later, Matloub R [5] presented an Al_0.9_Sc_0.1_N-based thin film bulk acoustic resonator (FBAR) with a resonant frequency of around 2.5 GHz and effective electromechanical coupling (*K_eff_*^2^) of 7.3%. Very recently, Shealy JB [6] demonstrated a 4.6 GHz XBAW resonator and 4.8 GHz filter based on single crystal Al_0.8_Sc_0.2_N superlattice with *K_eff_*^2^ of 10.7% and fractional bandwidth of 8.1%.

Despite its promising applications, it is still very challenging to design Al_1-x_Sc_x_N resonators operating at high frequencies. At high Sc concentration, the increase in the thermoelastic damping effect and the decrease in the stiffness for Al_1-x_Sc_x_N film would inevitably degrade the quality factor (Q) of the resonator [7]. Moreover, the mechanical and electrical losses of piezoelectric and electrode films increase dramatically with the increase in working frequency. Therefore, it is crucial to carefully design the BAW resonator structure and properly select the non-piezoelectric materials [8]. Asymmetrical electrodes [9,10], border frame structures and airgap edge reflectors [11] have been previously demonstrated as efficient methods to suppress the lateral acoustic wave leakage and also reduce the amplitude of the spurious modes. In addition, electrodes with high acoustic impedance and optimum thickness have been investigated to achieve the maximum *K_eff_*^2^ [12]. However, with the working frequency entering above 3 GHz and the adoption of much thinner electrodes, the disproportionately increased electrical loss, rather than conventional mechanical loss, would dominate in the energy loss, which can have a detrimental impact on the resonant performance. Therefore, the resonator structure, electrode material and thickness ratio of electrode to piezoelectric film should be carefully considered regarding both *K_eff_*^2^ and Q values.

In this paper, we optimized the Al_1-x_Sc_x_N-based BAW resonator structure for resonant performance at the high operating frequency by taking into account the geometric symmetry of the resonance region, border frame structure and thickness ratio of the piezoelectric film. On this basis, Al_0.7_Sc_0.3_N-based BAW resonators were fabricated and characterized, which exhibit a parallel resonance frequency of up to 4.75 GHz. Benefiting from the 30% Sc doping and high crystalline quality, the resonators achieve a *K_eff_*^2^ as high as 17.8% and a temperature coefficient of frequency (TCF) of −22.9 ppm/°C. Our results demonstrate the highly doped Al_1-x_Sc_x_N piezoelectric film as an ideal candidate for high-frequency BAW filter applications.

## 2. Bulk Acoustic Wave Resonator Design

**Figure 1:**
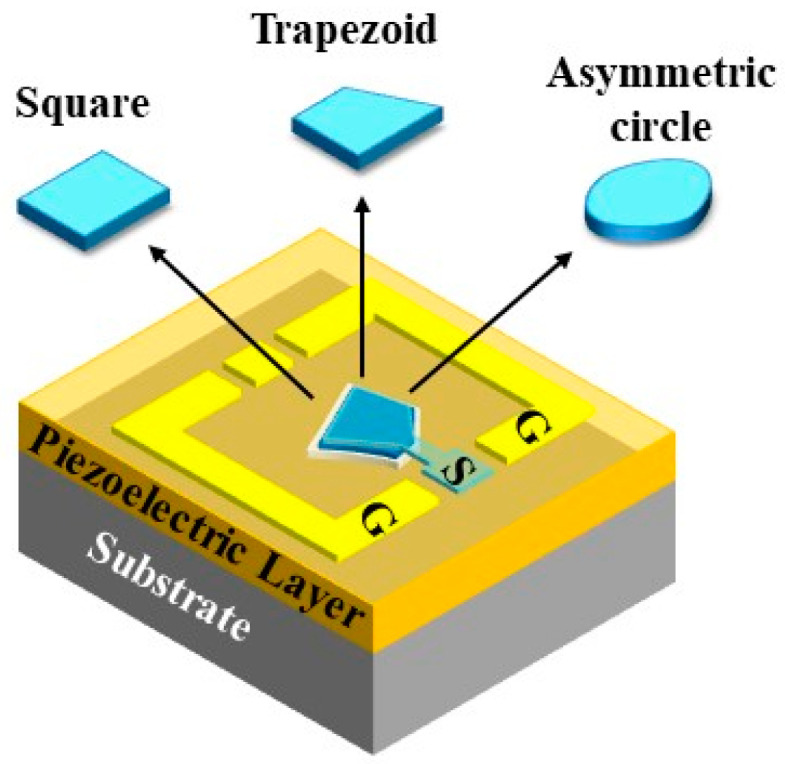
Schematic of BAW resonator. The geometrical shapes of the resonance region are square (two pairs of parallel edges), trapezoid (one pair of parallel edges) and asymmetric circle (non-parallel edge), respectively.

In our work, three-dimensional finite element method (FEM) simulations were used to evaluate the geometrical influence of resonant regions on resonance performance. As shown in Figure 1, AlN resonators were simulated with three types of shapes, including a square with two pairs of parallel edges, a trapezoid with one pair of parallel edge, and an asymmetric circle without any parallel edge. A three-layer stacked BAW resonator structure, including a 565 nm AlN piezoelectric film and 115 nm top and bottom electrodes, was designed and simulated with an air cavity underneath the resonant area. The resonant area was designed as 4783 μm^2^ to achieve 50 Ω impedance matching. The perfectly matched layer (PML) was set around the piezoelectric stack to eliminate acoustic wave reflection transversely.

**Figure 2:**
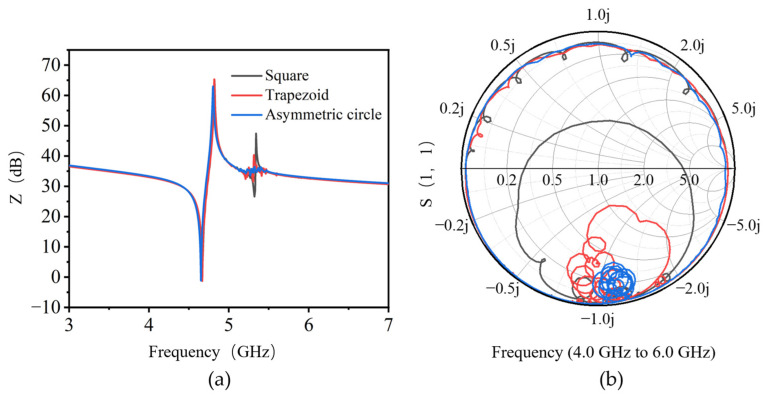
The simulated (**a**) impedance curves and (**b**) S(1,1) Smith charts of AlN BAW resonators with resonance shapes of square (two pairs of parallel edges), trapezoid (one pair of parallel edges) and asymmetric circle (non-parallel edge), respectively.

As can be seen from Figure 2a of simulated impedance characteristics, the series and parallel resonant frequencies of all three resonators, mainly determined by the thickness of the piezoelectric stacks, are around 4.65 GHz and 4.8 GHz, respectively. A strong parasitic resonance located around 5.3 GHz and several weaker spurious modes between series and parallel resonant frequencies are observed for the resonator with a symmetric square shape. It is interesting to find that these spurious modes are suppressed for the trapezoid-shaped resonator with one pair of parallel edges, and only small ripples are observed around 5.3 GHz for the completely asymmetric circle-shaped resonator. As further confirmed from the S(1,1) Smith chart shown in Figure 2b, compared with square and trapezoid-shaped resonance regions, the AlN BAW resonator with asymmetric circle shape indeed shows the weakest spurious modes. Since these modes are believed to arise from the parasitic lateral modes excited by Lamb waves, the asymmetric shapes would increase the path length of the lateral modes, which can reduce the coupling effects between the thickness-extensional (TE) mode and other lateral modes [13]. As a result, the standing waves are more attenuated as the symmetry of the active area is reduced. The adoption of asymmetric resonance area design can effectively suppress spurious modes and improve the resonance performance of BAW resonators.

**Figure 3:**
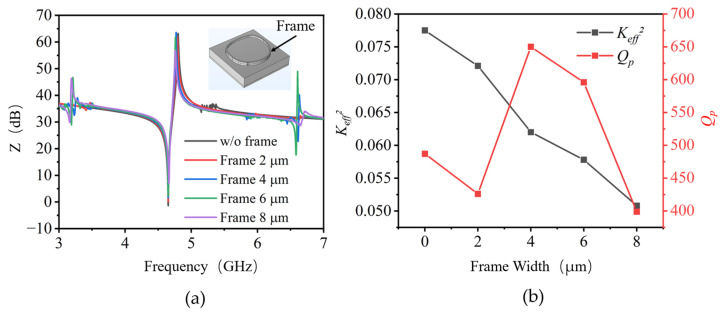
The simulated (**a**) impedance curves and (**b**) *Q_p_* and *K_eff_*^2^ dependence of AlN BAW resonators with different border frame widths.

The border frame structure, which is a thickened metal border structure above the edge of the top electrode, is usually designed to suppress the lateral acoustic wave leakage and enhance the quality factor (Q) of BAW resonators [11]. The performance of the AlN BAW resonators is further investigated by border frame structure introduction above top electrodes, as shown in the insert of Figure 3a. In the simulation, BAW resonators were constructed with a frame width ranging from 2 μm to 8 μm. The height of the frame was fixed at 200 nm, which was equal to the thickness of the Pad for fabrication process simplicity. Several spurious modes located between 5.1 GHz and 5.4 GHz are observed for AlN BAW resonators without a border frame structure, while they disappeared completely for that width frame structure with a frame width of 2–8 μm. Compared with a conventional structure, the AlN BAW resonators with frame structures exhibit much smoother resonance performance around the resonant frequency. However, two additional spurious modes emerge at approximately 3.2 GHz and 6.6 GHz, which are located far from the resonant frequency. Furthermore, it can be found in Figure 3b that with the frame width increasing, the *K_eff_*^2^ decreases proportionally while *Q_p_* fluctuates between 399 and 650. It is worth noting that 33.5% and 22.4% *Q_p_* improvement were observed for the resonators with frame widths of 4 μm and 6 μm, respectively. The specific boundary created by the appropriate border frame structure can prevent the acoustic waves from propagating through lateral directions so that more energy is confined in the resonance region, resulting in lower acoustic loss and higher Q values. Accordingly, AlN BAW resonators with suitable border frame structures are desirable for acoustic energy confinement.

**Figure 4:**
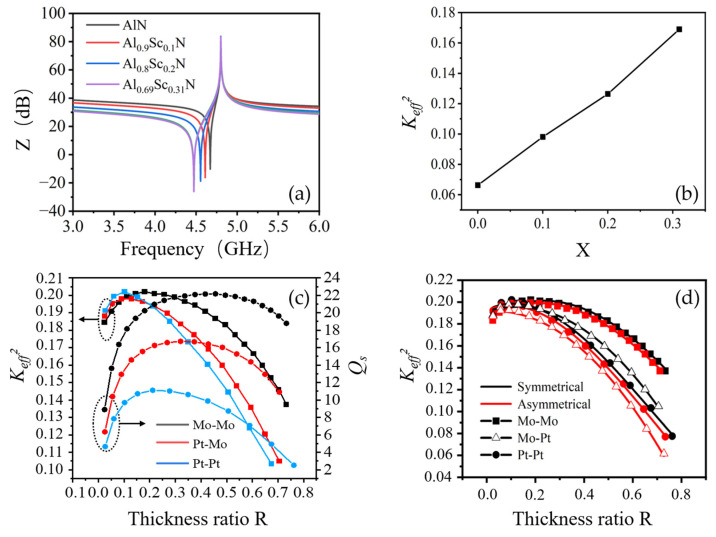
Effects of Sc concentration and thickness ratio on resonator performance. (**a**) simulated impedance curves and (**b**) *K_eff_*^2^ of Al_1-x_Sc_x_N BAW resonators with different Sc concentrations. (**c**) *K_eff_*^2^ and *Q_s_* of Al_0.69_Sc_0.31_N resonators with different thickness ratio (R) and thickness-symmetrical electrode structure. (**d**) *K_eff_*^2^ of Al_0.69_Sc_0.31_N resonators with symmetrical and asymmetrical electrode structure.

The resonant characteristics of Al_1-x_Sc_x_N BAW resonators with different Sc concentrations were also investigated. Material parameters of Al_1-x_Sc_x_N piezoelectric films with different Sc concentrations used for the simulation are obtained by first-principles calculation, and the acoustic impedance and velocity of Pt and Mo were obtained from ref. [12], as shown in Table 1. The thickness of Al_1-x_Sc_x_N films is varied for different Sc content to keep the parallel frequency of BAW resonators fixed at 4.8 GHz, and the thickness of the bottom Pt electrode and top Mo electrode is fixed at 100 nm. As Sc concentration is increased from 0% to 31%, the thickness of Al_1-x_Sc_x_N films is decreased from 390 nm to 266 nm, which is caused by the lower stiffness and the consequent lowered longitudinal acoustic velocity of the increasing Sc concentration [14]. More importantly, the series frequency extends from 4.67 GHz to 4.47 GHz (see Figure 4a), which corresponds to the increase in *K_eff_*^2^ from 6.63% to 16.9% (see Figure 4b). These observations indicate that the bandwidth of subsequent BAW filters would be increased dramatically.

Although the thickness ratio between electrodes and piezoelectric layer was previously investigated for resonator *K_eff_*^2^ optimization [12], the thinning of electrodes towards sub-100 nm as working frequency approaching above 5 GHz would need additional considerations on the quality factor Q of BAW resonators. Due to the increased electrical loss caused by the large ohmic resistance, Q would significantly decrease, which can further deteriorate the isolation performance of BAW filters. Therefore, the dependence of both *K_eff_*^2^ and *Q_s_* of Al_0.69_Sc_0.31_N resonators on the thickness ratio R (top and bottom electrodes thickness/total piezoelectric stack thickness) and electrode materials was investigated for thickness-symmetrical electrode structures, as shown in Figure 4c. The thickness of Al_0.69_Sc_0.31_N film decreases accordingly as the increase in R maintains the parallel frequency of 4.8 GHz.

As can be seen, the *K_eff_*^2^ of Al_0.69_Sc_0.31_N resonators increases and reaches a characteristic peak slightly above 20% when R is around 0.1 and 0.18 for Pt-Pt and Mo-Mo electrodes, respectively. The increase in *K_eff_*^2^ is due to the improved match in the distribution of the acoustic standing wave to the linear distribution of applied electric potential [8]. As R continues to increase, *K_eff_*^2^ begins to decrease as more non-piezoelectric electrode material occupies the piezoelectric stacks, which eventually deteriorates the coupling efficiency. Since the acoustic velocity of Mo is higher than that of Pt, the peak thickness ratio R of Mo-Mo electrodes is relatively larger than that of Mo-Pt and Pt-Pt. The peak *K_eff_*^2^ of Pt-Pt electrodes is slightly higher than that of Mo-Mo due to the higher acoustic impedance of Pt, resulting in the larger acoustic impedance mismatch between electrode and piezoelectric films [15]. The *K_eff_*^2^ performance of Pt-Mo electrodes is located in between. The *Q_s_* of the Al_0.69_Sc_0.31_N resonators share a similar phenomenon. The *Q_s_* of Mo-Mo and Pt-Pt electrodes reach their maximum at R about 0.45 and 0.2, respectively, where the electrical loss and mechanism loss are balanced. The electrical loss dominates *Q_s_* as the thickness of electrodes decreases, while mechanism loss begins to dominate beyond the peak. The optimum electrode materials and thickness ratio (R) should be carefully designed based on the practical requirement of working frequency, bandwidth and out-of-band rejection.

In addition, the *K_eff_*^2^ of Al_0.69_Sc_0.31_N resonators with symmetrical and asymmetrical electrode thickness was studied for Mo-Mo, Pt-Pt and Mo-Pt electrodes, as shown in Figure 4d. The thickness of the top and bottom electrodes is equal for the symmetrical structure, while the thickness of the bottom electrode is twice as large as that of the top one for the asymmetrical one. The *K_eff_*^2^ of the asymmetrical electrode exhibits a similar trend to that of the symmetrical one; however, the value of *K_eff_*^2^ is slightly lower due to asymmetric acoustic energy distribution along the thickness direction. Moreover, high-order resonant modes will be excited for asymmetrical electrodes, which degrades the *K_eff_*^2^ of the fundamental one [15].

According to the above simulation results, Al_1-x_Sc_x_N resonators with a Sc concentration of 30% were designed for *K_eff_*^2^ above 15%. A thickness-symmetrical Mo-Pt electrode structure with a thickness ratio of about 0.3 was designed to balance the *K_eff_*^2^ and *Q_s_* performance. The asymmetric circular resonant shape and border frame structure were used for lateral spurious modes suppression.

## 3. Experimental Results and Discussion

**Figure 5:**
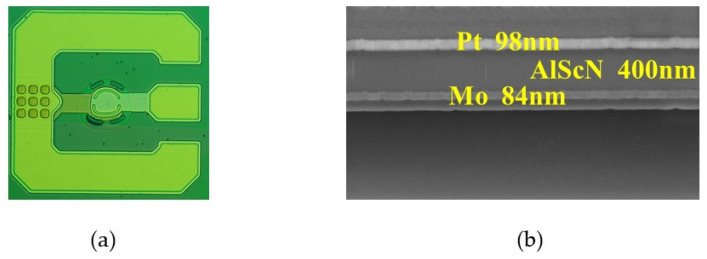
The fabricated Al_0.7_Sc_0.3_N resonator and piezoelectric stacks. (**a**) Optical image of fabricated resonator with asymmetrical-circular active area and border frame structure. (**b**) Cross-sectional scanning electron microscope (SEM) image of piezoelectric stacks above air cavity.

As shown in Figure 5a, Al_0.7_Sc_0.3_N BAW resonators were fabricated on a 4-inch wafer using a micro-electromechanical systems sacrificial process. 400 nm Al_0.7_Sc_0.3_N piezoelectric film was deposited using Evatec CLN200 sputtering system. The longitude piezoelectric coefficient (d_33_) of Al_0.7_Sc_0.3_N film was determined as high as 14.96 pC/N using the Berlincourt method PIEZOTEST PM300, which is nearly three times larger than that of pure AlN film. 90 nm Mo and Pt were deposited as bottom and top electrodes. Ti/Au with 5 nm/200 nm was deposited and patterned as pad and frame structure at the same time. Two face-to-face semicircles with a frame width of 6 μm and height of 200 nm were fabricated as border frame structures for better process controllability. A 1 μm amorphous silicon was used as a sacrificial layer and finally selectively etched by XeF_2_. About 100 nm SiO_2_ was used as a protecting and supporting layer underneath the bottom electrode. The fabricated Al_0.7_Sc_0.3_N BAW resonator has an asymmetrical circular shaped resonant area, with 400 nm Al_0.7_Sc_0.3_N piezoelectric film, 84 nm Mo bottom electrode and 98 nm Pt top electrode above the air cavity (see Figure 5b). The measured thickness ratio R is about 0.31, which is in good agreement with the designed thickness ratio of 0.3.

**Figure 6:**
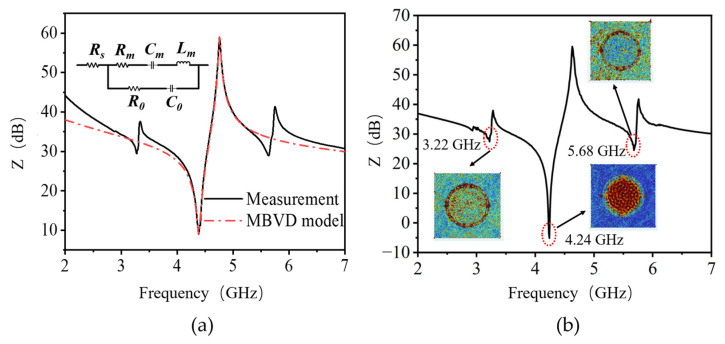
(**a**) Measured and MBVD-fitted curves of the fabricated Al_0.7_Sc_0.3_N BAW resonator. (**b**) Simulated impedance curve and inserted mechanical displacement distributions of Al_0.7_Sc_0.3_N BAW resonator by FEM.

Figure 6a shows the impedance characteristics of the fabricated Al_0.7_Sc_0.3_N resonator. The device exhibits a fundamental thickness mode with a serial resonance frequency (*f_s_*) of 4.38 GHz and a parallel resonance frequency *f_p_* of 4.75 GHz. The frequency can be further precisely tuned by focus ion beam (FIB) trimming of piezoelectric stacks for specific applications [16]. The *K_eff_*^2^ is calculated by the following equation [14]:

(1) $$K_{eff}{}^{2}=\frac{\pi^{2}}{4}\times \frac{f_{s}}{f_{p}}\times \frac{f_{p}-f_{s}}{f_{p}}$$

*K_eff_*^2^ as high as 17.8% was obtained for the fabricated resonator. The superior *K_eff_*^2^ of the fabricated resonator is directly attributed to the significantly improved piezoelectric response of 30% Sc doped Al_0.7_Sc_0.3_N piezoelectric film. The *Q_s_* of 107, *Q_p_* of 139 and *Q_max_* of 194 are extracted based on the following equation [17]:

(2) $$Q_{s,p}=\frac{f_{s,p}}{2}|\frac{d\phi_{z}}{df}|$$

(3) $$Q_{max}=\frac{2\pi f\left|S_{11}\right|\tau \left(f\right)}{1-{\left|S_{11}\right|}^{2}}$$

where $\phi_{z}$ is the phase of impedance and τ is the measured group delay. FoM (*K_eff_*^2^·*Q_max_*) of 34.5 is achieved for the fabricated Al_0.7_Sc_0.3_N resonator. As shown, *Q_p_* and *Q_s_* are mainly determined by the mechanical loss and electrical loss separately. With the increase in Sc concentration, the crystalline quality of Al_1-x_Sc_x_N film deteriorates dramatically due to larger lattice mismatch, higher intrinsic stress and the formation of abnormally oriented grains, which is believed to be the main reason for the relatively low *Q_p_* [17]. The high ohmic resistance of sub-100 nm thick electrodes also leads to a sharp increase in electrical loss that lowers *Q_s_*. Compared with previously reported works summarized in Table 2, the fabricated Al_0.7_Sc_0.3_N resonator exhibits balanced *K_eff_*^2^ and Q resonance performance in the SHF band, demonstrating great potential for high-frequency and wideband applications.

The impedance characteristics in Figure 6a are fitted by the modified Butterworth-Van Dyke (MBVD) model, including static capacitor *C*_0_, motional capacitor *C_m_*, motional inductor *L_m_*, motional resistance *R_m_*, dielectric loss *R*_0_ and ohmic resistance *R_s_* [21]. The fitting shows a reasonably good agreement with the fundamental resonant mode of the measurement results, and the extracted parameters are listed in Table 3. It is worth noting that the ohmic loss *R_s_* is 1.75 Ω, larger than the mechanical and dielectric losses due to the ultra-thin electrodes below 100 nm, which is consistent with the Q values discussed above.

In addition to the fundamental thickness mode, two spurious modes located around 3.27 GHz and 5.63 GHz were observed in the impedance curve. The unoptimized border frame structure is believed to be responsible for this. The impedance curve and mechanical displacement distributions of the Al_0.7_Sc_0.3_N resonator with a border frame structure that is 4 μm wide and 230 nm high were simulated in Figure 6b. Similar to the measurement results, three resonance modes, including the fundamental mode at 4.24 GHz and two spurious modes at 3.22 GHz and 5.68 GHz, are observed from the impedance curve. More importantly, as shown in the insert of Figure 6b, the mechanical displacement distribution for the fundamental resonance mode is confined within the active area, while those of the two spurious modes are around the perimeter of the active area, which matches well with the frame structure. The geometrical differences in the frame structure between simulation and fabrication may be induced by the process variations and metal edge peeling during the sacrificial release step. Therefore, the thickness and width of the border frame structure should be further optimized considering the process variation for sub-resonance elimination [22].

**Figure 7:**
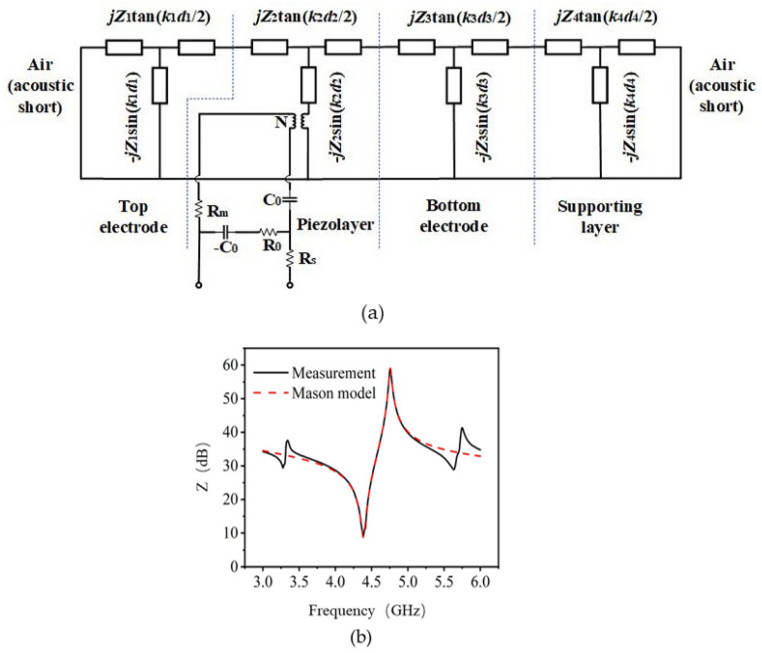
(**a**) Equivalent mason circuit model of the fabricated Al_0.7_Sc_0.3_N BAW resonator. (**b**) The measurement and Mason model fitted impedance curves of Al_0.7_Sc_0.3_N resonators.

Mason model was used to evaluate key material properties of Al_0.7_Sc_0.3_N piezoelectric material [23]. A one-dimensional equivalent circuit, including a piezoelectric layer, supporting layer, and top and bottom electrodes, was built up. As shown in Figure 7a, *Z_i_*, *k_i_* and *d_i_* are the characteristic acoustic impedance, wavenumber and thickness of each layer, respectively. $N=\sqrt{k_{2}d_{2}/2\pi fK_{t}{}^{2}C_{0}Z_{2}}$, where *K_t_*^2^ is the electromechanical coupling factor of Al_0.7_Sc_0.3_N piezoelectric film. The thicknesses were obtained from SEM measurements. Material parameters are extracted using the following equation [23]:

(4) $$Z=\rho v_{a}$$

(5) $$v_{a}=\sqrt{\frac{c_{33}}{\rho }}$$

(6) $$K_{t}{}^{2}=\frac{e_{33}{}^{2}}{c_{33}\varepsilon_{r}\varepsilon_{0}}$$

(7) $$C_{0}=\frac{\varepsilon_{r}\varepsilon_{0}A}{d_{2}}$$

where ρ is the mass density, $v_{a}$ is the acoustic velocity, $c_{33}$ is the elasticity constant, $e_{33}$ is the piezoelectric stress constant, $\varepsilon_{r}$ is the relative dielectric permittivity, and A is the active area of the resonator. The extracted results are listed in Table 4. $e_{33}$ of Al_0.7_Sc_0.3_N film is 2.49 C/m^2^, significantly improved compared with pure AlN film (1.46 C/m^2^) [24], while the acoustic velocity of Al_0.7_Sc_0.3_N film decreases to 8418 m/s, 20% lower than that of pure AlN, which matches reasonably well with calculation results.

**Figure 8:**
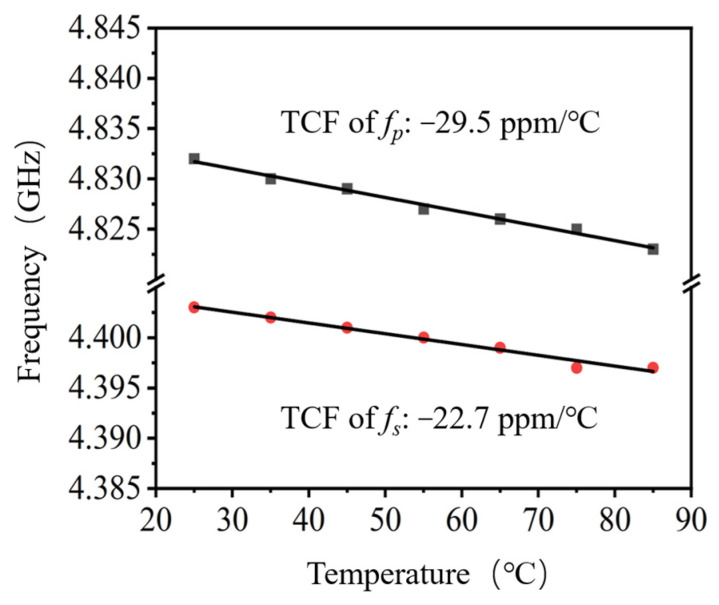
Measured *f_s_* and *f_p_* TCF of the fabricated Al_0.7_Sc_0.3_N BAW resonator.

The temperature coefficient of frequency (TCF) characterizes the thermal frequency stability of resonators. The resonant frequency response of the Al_0.7_Sc_0.3_N device at different temperatures ranging from 25 °C to 85 °C was measured, as shown in Figure 8. The TCFs of *f_s_* and *f_p_* are extracted to be −22.7 ppm/°C and −29.5 ppm/°C, respectively. Both values are slightly higher than that of pure AlN-based FBAR (about −25 ppm/°C) [25], which is believed to be caused by the deterioration of the temperature coefficient of stiffness due to the high concentration of Sc doping [26].

## 4. Conclusions

In this work, we designed and fabricated high-frequency BAW resonators based on Al_1-x_Sc_x_N piezoelectric film with a high Sc doping concentration of 30%. Asymmetric resonator shapes and border frame structures were designed for lateral modes suppression and acoustic energy confinement. The thickness and symmetry of piezoelectric stacks were investigated and optimized regarding *K_eff_*^2^ and Q to achieve balanced, resonant performance. 4.75 GHz BAW resonators were successfully fabricated based on Al_0.7_Sc_0.3_N piezoelectric film, with *K_eff_*^2^ as high as 17.8% and TCF of −22.7 ppm/°C and −29.5 ppm/°C at *f_s_* and *f_p_*, respectively. The superior *K_eff_*^2^ of fabricated Al_1-x_Sc_x_N resonator exhibits significant advantages of BAW filters in wireless communication applications operating in the emerging SHF band.

## Figures and Tables

**Table 1:** Material parameters of simulation [12].

Material	Acoustic Impedance [10^6^ kg/m^2^s]	Acoustic Velocity [m/s]	*K_t_^2^*
AlN	35.01	10,544	0.068
Al_0.9_Sc_0.1_N	33.35	9823	0.098
Al_0.8_Sc_0.2_N	31.07	8992	0.125
Al_0.69_Sc_0.31_N	29.20	8216	0.163
Pt	69.00	3260	----
Mo	63.10	6250	----

**Table 2:** Resonant performance comparison with other reports.

Piezoelectric Materials	Frequency (*f_p_*) (GHz)	*K_eff_* ^2^	*Q_max_*	Ref.
Al_0.8_Sc_0.2_N	4.588	14.5%	318	[18]
Al_0.7_Sc_0.3_N	3.17	18.1%	210	[19]
Al_0.7_Sc_0.3_N	3.17	11.4%	572	[20]
Al_0.7_Sc_0.3_N	4.756	17.8%	194	This work

**Table 3:** MBVD model parameters.

*C*_0_[pF]	*C_m_*[pF]	*L_m_*[nH]	*R_m_*[Ω]	*R*_0_[Ω]	*R_s_*[Ω]
0.82	0.15	9.09	1.10	0.80	1.75

**Table 4:** Film parameters Extracted from the Mason Model.

Material	*K_t_* ^2^	*ε_r_*	*ρ*(kg/m^3^)	*V_a_*(m/s)	*C*_33_(Gpa)	*e*_33_(C/m^2^)
Al_0.7_Sc_0.3_N	16.37%	13.89	3485	8418	247	2.23

## Data Availability

Data are contained within the article.
